# Role of upper airway ultrasound in airway management

**DOI:** 10.1186/s40560-016-0174-z

**Published:** 2016-08-15

**Authors:** Adi Osman, Kok Meng Sum

**Affiliations:** 1 Department of Trauma & Emergency, Hospital Raja Permaisuri Bainun, Ipoh, Perak Malaysia; 2 Department of Anesthesiology & Intensive Care, Beacon International Specialist Centre, Selangor, Malaysia

**Keywords:** Upper airway ultrasound, Point of care ultrasound, Airway management

## Abstract

Upper airway ultrasound is a valuable, non-invasive, simple, and portable point of care ultrasound (POCUS) for evaluation of airway management even in anatomy distorted by pathology or trauma. Ultrasound enables us to identify important sonoanatomy of the upper airway such as thyroid cartilage, epiglottis, cricoid cartilage, cricothyroid membrane, tracheal cartilages, and esophagus. Understanding this applied sonoanatomy facilitates clinician to use ultrasound in assessment of airway anatomy for difficult intubation, ETT and LMA placement and depth, assessment of airway size, ultrasound-guided invasive procedures such as percutaneous needle cricothyroidotomy and tracheostomy, prediction of postextubation stridor and left double-lumen bronchial tube size, and detecting upper airway pathologies. Widespread POCUS awareness, better technological advancements, portability, and availability of ultrasound in most critical areas facilitate upper airway ultrasound to become the potential first-line non-invasive airway assessment tool in the future.

## Background

Numerous studies and reports have described various roles of ultrasound imaging in airway management in recent years. It helps in rapid assessment of the airway anatomy in the operating theater, intensive care unit, emergency department, and even in remote/austere environment. This article highlights briefly the review of various published studies and case reports on the role of upper airway ultrasound in airway assessment and management.

## Applied sonoanatomy of the upper airway

Basic comprehension of ultrasound physics, transducer selection, body habitus, and probe orientation and better understanding of airway anatomy contribute to accuracy of ultrasound interpretation.

**Fig. 1:**
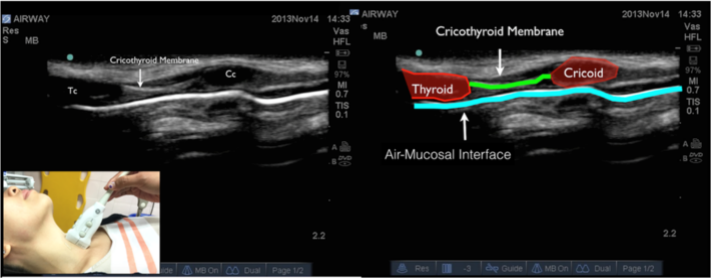
Cricoid cartilage, thyroid cartilage, and cricothyroid membrane in longitudinal plane. *Cc* cricoid cartilage, *Tc* thyroid cartilage

**Fig. 2:**
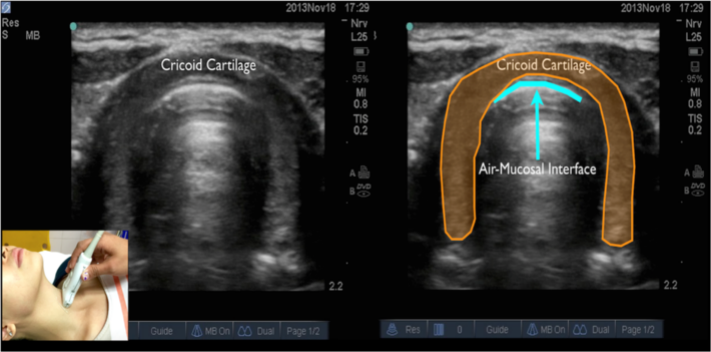
Cricoid cartilage in transverse plane

**Fig. 3:**
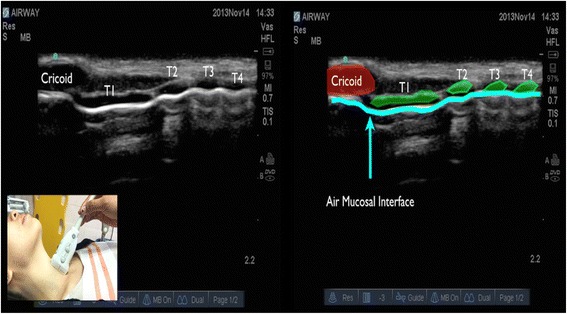
Cricoid cartilage and tracheal cartilage in longitudinal plane is seen as a “string of beads.” *T1* first tracheal cartilage, *T2* second tracheal cartilage, *T3* third tracheal cartilage, *T4* fourth tracheal cartilage

**Fig. 4:**
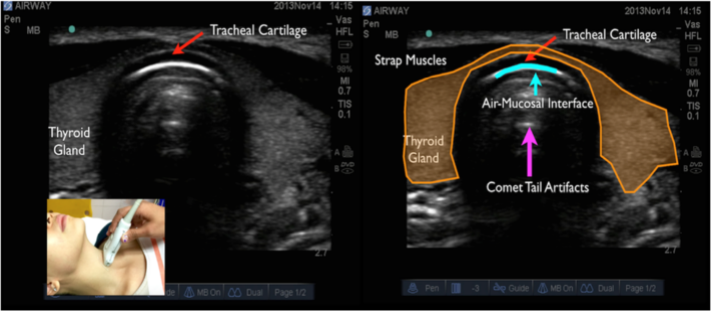
Tracheal cartilage in transverse plane

In longitudinal plane, the hypoechoic appearance of cricoid cartilage is seen as a bump or hump (Fig. 1) and in transverse plane as an oval hypoechoic structure (Fig. 2). Tracheal cartilage in longitudinal plane is seen as a “string of beads” (Fig. 3) and inverted *U* in the transverse plane (Fig. 4). A linear hyperechoic line seen posteriorly on transverse and longitudinal plane of trachea is formed by reverberation artifacts from air-mucosal interface [1, 2].

**Fig. 5:**
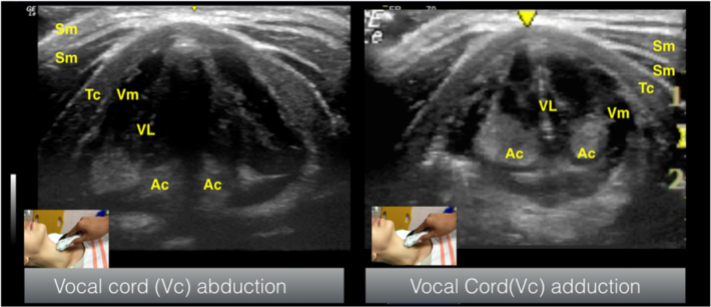
Vocal cord seen in transverse view. *Sm* sternocleidomastoid muscle, *Tc* thyroid cartilage, *VM* vocalis muscle, *VL* vocalis ligament, *AC* arytenoid cartilage

**Fig. 6:**
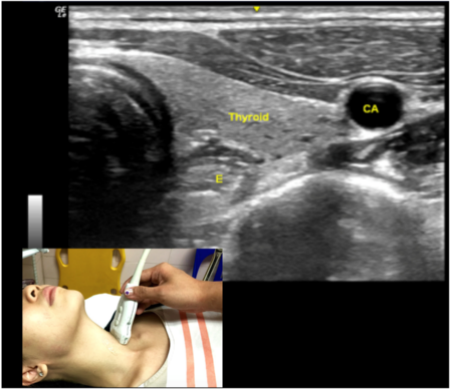
Esophagus is seen using transverse plane at the level of the first and second tracheal cartilage. *E* esophagus, *CA* carotid artery

Vocal cord is best seen using transverse plane through the thyroid cartilage as a window. The hyperechoic appearance of the vocal ligaments delineates the vocal cord (Fig. 5). Esophagus is seen using transverse plane at the level of the first and second tracheal cartilage, located posterior to the left thyroid nodule (Fig. 6). Visible peristaltic movement can be seen inside the esophageal lumen by swallowing activity.

## Airway size and prediction of endotracheal tube (ETT) size

There is growing academic interest in the ability of ultrasound to determine airway size and estimate appropriate ETT size clinically.

Ultrasound is a reliable tool for assessment of the subglottic airway. It is validated against the magnetic resonance imaging [3] and computed tomography scan [4]. The advantages of ultrasound to other radiological methods in the assessment of the subglottic airway lie in the fact that ultrasound requires minimal training [3] and do not require complete immobility or sedation.

There is good correlation between subglottic transverse diameter measured by ultrasound and the outer ETT diameter [5–7]. ETT size, both cuffed and uncuffed, can be measured accurately using ultrasound [5]. Ultrasound is superior to age-based [5, 6] and height-based formula [5] in estimating ETT size.

Shibasaki et al. measured the subglottic diameter at the lower edge of the cricoid cartilage and standardized respiratory parameters during measurement (using muscle relaxation without the use of PEEP) and found that age- and height-based formula can only predict accurately 35 % of cuffed ETT size and 60 % of uncuffed tube size compared to ultrasonography (98 and 96 %, respectively) [5].

The superiority of ultrasound compared to standard age-based formula is supported by Bae et al. [6] and Schramm et al. [8], except the ability to predict correct ETT size varies between the two studies 60 % [6] and 48 % [8], respectively. Kim et al. found good correlation between outer diameter of ETT at subglottic level and the actual outer diameter of the ETT and suggested a formula to choose the appropriate ETT size in children [7].

## Difficult laryngoscopy

Ultrasound images of the infrahyoid parameters correlate well with computed tomography scan [9]. Hui et al. demonstrated that the inability to visualize the hyoid bone using sublingual ultrasound predict difficult intubation with a high sensitivity and specificity. This method has a high-positive likelihood ratio of 21.6 and moderate-negative likelihood ratio of 0.28 [10].

Wotjzak et al. found that the hyomental distance ratio (the distance between hyoid bone and mandibular mentum in the neutral to the hyperextended neck position) in the morbidly obese patients can be a sensitive predictor of difficult laryngoscopy. Patients with difficult intubation have shorter hyomental distance ratio of 1–1.05 as opposed to those belonging to the easy intubation group (1.12–1.16) [11].

Ezri et al. showed that mean pretracheal tissue of 28 mm (standard deviation (SD) 2.7 mm) at the level of the vocal cords in obese patients indicate difficult laryngoscopy compared to 17.7 mm (SD 1.8 mm) in easy ones [12]. This technique is not reproducible by Komatsu et al. using similar methodology in different population [13]. Adhikari et al. demonstrated in a pilot study that anterior neck thickness at the level of the hyoid bone and thyrohyoid membrane (more than 2.8 cm) as compared to the vocal cords is a better predictor for difficult laryngoscopy [14]. Pinto et al. in a recent study found similar findings using anterior neck thickness at the level of the thyrohyoid membrane [15].

Preliminary findings showed promising results, but most of these studies were pilot studies and were limited by small study samples. More systematic studies and level one evidence are needed before this modality can be validated for routine screening.

## Airway device placement and depth

### Endotracheal tube (ETT) confirmation

**Fig. 7:**
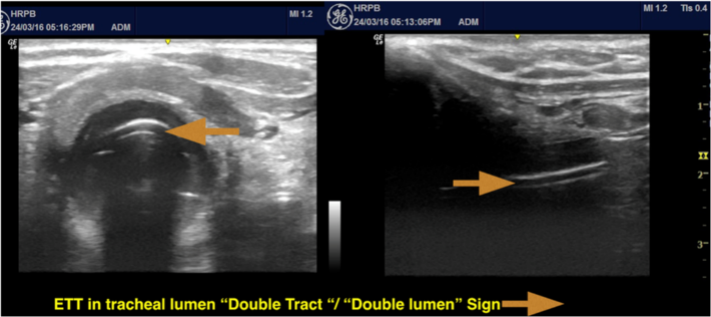
Transverse and longitudinal view of ETT in trachea, seen as “double tract”/“double lumen” sign

Although many techniques have been recommended to verify ETT location, there is no single confirmatory method that is ideal in every situation [16]. Capnography is considered the standard of care for the primary verification of ETT location. Upper airway ultrasonography can also be advantageous in situations involving cardiovascular arrest, bronchoconstrictions or circumstances in which capnography or end-tidal carbon dioxide measurement (ETCO2) may be faulty [1, 17, 18]. ETT position in trachea is seen as two hyperechoic lines which is described as “double tract” or “double lumen” sign [1, 3] (Fig. 7)

.

Chou et al. utilizes a convex transducer in the suprasternal notch window, tracheal rapid ultrasound exam (T.R.U.E.), for diagnosing esophageal intubation and demonstrated 98.9 % sensitivity and 94.1 % specificity for ETT utilizing this static transtracheal approach [19].

Adi et al. showed that bedside upper airway ultrasound correlates well with waveform capnography. This research also shows very good agreement with kappa value of 0.85, between bedside upper airway ultrasound and waveform capnography, and a fast mean confirmation time of 16.4 s with a standard deviation of 7.3 s [1].

Meta-analysis by Chou et al. suggested that in situations where capnography may be unreliable, ultrasonography can be a valuable adjunct in this aspect of airway assessment because ultrasonography has high diagnostic value for identifying esophageal intubation with optimal sensitivity and specificity [2].

### Endotracheal tube (ETT) depth

Sitzwohl et al. found that auscultation and chest rise during clinical assessment failed to detect up to 55 % of endobronchial intubations [20]. A cadaver study by Uya et al. showed that novice sonographers could accurately identify a saline-inflated ETT cuff at the level of the suprasternal notch [21]. Tessaro et al. using tracheal rapid ultrasound saline test (TRUST) technique suggests that tracheal ultrasonography using a saline-filled ETT cuff can accurately and rapidly distinguish between correct endotracheal versus endobronchial tracheal tube positions in children [22].

### Laryngeal mask airway (LMA) confirmation

Wojtczak et al. demonstrates that the replacement of air with saline in ETT or LMA cuffs, and the use of contrast agents enable detection of cuffs in the airway. It also allows visualization of the surrounding structures and tissues as the ultrasound beam can be transmitted through the fluid—filled cuffs without being reflected from air-mucosal interface [23].

Kim et al. conducted an observational study in children comparing upper airway ultrasound versus fiberoptic bronchoscopy to evaluate the incidence of LMA malposition. Based on graded sonographic arytenoid cartilage elevation on transverse plan (Fig. 5) comparing with fiberoptic bronchoscopy (as an indicator of LMA malrotation), they suggest ultrasound could detect LMA malrotation easily in pediatric patients. This study demonstrated that ultrasound have a high sensitivity of 93 % and specificity of 82 % with accuracy of 87 % (95 % CI, 79–93 %) [24].

## Use of upper airway ultrasound for percutaneous cricothyroidotomy

Cricothyroidotomy is a life-saving procedure in the “cannot intubate cannot ventilate” situation, but the cricothyroid membrane may not be easily identified by the landmark techniques [25–27]. Mallin et al. found that the cricothyroid membrane landmark before and after a difficult airway simulation is similar. This further illustrates the usefulness of marking the cricothyroid membrane before an anticipated difficult intubation [28].

Nicholls et al. studied the usefulness of ultrasound as a guide to identify the cricothyroid membrane using a standardized technique in cadavers before allowing two emergency physicians to obtain sonographic images of 50 patients in the emergency department. The mean time taken to visualize the cricothyroid membrane was less than 25 s with shallow learning curve [29]. Curtis et al. demonstrates the feasibility of real-time ultrasound-guided cricothyroidotomy using a bougie-assisted cricothyroidotomy in cadavers. The median time to identify the cricothyroid membrane was less than 4 s, and to completion of procedure was less than half a minute with high success rate [30].

Randomized trial by Siddiqui et al. comparing digital palpation versus ultrasonography by non-experienced ultrasound user in cadaver model showed that injuries to the airway were three times lower in the ultrasound-guided group even in cadavers with distorted neck anatomy, although it took longer to complete the procedure (196 vs 110 s) [31].

## Use of upper airway ultrasound for percutaneous dilational tracheostomy (PDT)

Ultrasound provides good anatomical landmark for PDT. It also allows clinician to estimate appropriate tracheostomy tube size and length [32] and avoid anterior neck structures [33, 34] and posterior tracheal wall injuries [35].

Preprocedural airway ultrasound improves safety of PDT [33, 34, 36]. Studies found that about one in four patients required a change in PDT puncture site after ultrasound assessment [34].

Brueggeney et al. illustrates the feasibility of real-time in-plane ultrasound-guided PDT in facilitating successful tracheal puncture and wire insertion in cadaver [37]. The Traditional landmArk versus ultRasound Guided Evaluation Trial (TARGET) Study [38] and Dinh et al. [39] found that real-time ultrasound guidance improve the success rate of the first PDT attempt and puncture accuracy compared to traditional anatomical landmark.

Gobatto et al. in the TRACHUS randomized controlled trial looking at procedure failure and major complications suggest that ultrasound-guided PDT is as good as bronchoscopy-guided PDT in critically ill patients [40].

When compared to the conventional landmark methods, ultrasound-guided PDT scored a higher success rates with shorter time to successful cannulation [41].

## Prediction of postextubation stridor

Ultrasound measurement of air column width at the level of the vocal cords has the potential to predict postextubation stridor [42, 43]. Ding et al. compared air column width before and after ETT cuff deflation, which represent the amount of air passing through the vocal cords. This research found limited air column width after deflation of ETT cuff and limited air column width difference (postdeflation air column width − predeflation air column width) to be associated with postextubation stridor [42].

Sutherasan et al. using the same methodology found that the mean air column width difference in patients with and without postextubation stridor is 1.99 and 1.08 mm, respectively, and suggest a cutoff value of 1.6 mm to predict postextubation stridor. This value carries a sensitivity of 70 % and specificity of 70 % with a high negative predictive value of 92 % and low positive predictive value of only 32 % [43].

This result is not reproducible using similar technique by Mikaeili et al. who observed low sensitivity, specificity, positive predictive and negative predictive value of air width column difference, and post-ETT cuff deflation air column width [44].

Further studies with bigger sample size are required to obtain more reliable cutoff points to determine the value of ultrasound in predicting postextubation stridor.

## Predicting the size of a left double-lumen bronchial tube

Sustic et al. described a strong correlation between outer tracheal width measured using ultrasound at the level just above the sternoclavicular junction and that of internal tracheal width and left mainstem bronchus size measured by computed tomography scan. The ultrasonographic measurement of outer tracheal width may be used to select the size of a left-sided double-lumen tube [4].

## Evaluation of the epiglottis

The application of upper airway ultrasound for evaluation of epiglottis is promising, and preliminary studies to evaluate the epiglottis have shown success. Werner et al. reported the utility of ultrasound to image the epiglottis and determine the average range of epiglottis diameter for men and women (average patient age was 35.2 ± 8.1 years). The epiglottic thickness was 2.39 (±0.15 mm), which was greater in men (2.49 ± 0.13 mm) than in women (2.34 ± 0.13 mm) [45]. Ko et al. measured the anteroposterior diameter of the epiglottis and showed that there was significant difference between patients with epiglottitis in the emergency department setting and healthy volunteers [46]. Hung et al. reported a case study using transducer placed in longitudinal plane of thyrohyoid membrane in patients diagnosed with epiglottitis and described the hyperechoic appearance of thickened epiglottis in relation to the acoustic shadowing of the hyoid bone as an “alphabet P sign” [47].

Limitations of these studies are the small sample size, and further research need to determine the normal population based on average epiglottis thickness.

## Upper airway ultrasound educational learning curve

Upper airway ultrasound is operator-dependent and requires proper training to become proficient in its use. Gottlieb et al. demonstrated high sensitivity and specificity of the four-step (4S) technique to confirm ETT placement on thin adult human cadaver model in both expert and novice (emergency medicine resident) ultrasound user [48].

Uya et al. reported short didactic training session on emergency medicine fellows focused on upper airway ultrasound (20 min), followed by a 30-min practice session on limited ultrasound baseline knowledge. ETT placement was correctly identified in 39 of 40 scans, with a sensitivity of 96 % (23 of 24) for identifying tracheal location. In those with tracheal intubations, tube depth was correctly identified in 22 of 23 scans [21].

Chenkin et al. found that after a brief online tutorial (10 min) and only two practice attempts, emergency physicians were proficient to interpret ultrasound clips of esophageal and endotracheal intubation (90.9 % one practice attempt and 100 % after two practice attempts) [49].

Clinician-performed upper airway ultrasound shows positive signs of being an effective tool for airway management, but further research is needed to validate the educational learning curve when compared with the current standard.

## Conclusions

Usage of upper airway ultrasound is useful in dealing with critically ill patients especially in airway management because of its portability, non-invasiveness, cost-effectiveness, and reproducibility. With promising and increasing numbers of evidence exists, there is a potential for the incorporation of upper airway ultrasound into the future standard of care airway assessment, monitoring, and imaging modalities.

## Abbreviations

ETT, endotracheal tube; LMA, laryngeal mask airway; POCUS, point of care ultrasound; Tc, thyroid cartilage; Cc, cricoid cartilage; VM, vocalis muscle; VL, vocalis ligament; AC, arytenoid cartilage; PDT, percutaneous dilational tracheostomy; SD, standard deviation
